# Pattern of Antibiotic Consumption in Two Italian Production Chains Differing by the Endemic Status for Porcine Reproductive and Respiratory Syndrome

**DOI:** 10.3389/fvets.2022.840716

**Published:** 2022-03-28

**Authors:** Paolo Trevisi, Laura Amatucci, Roberta Ruggeri, Costanza Romanelli, Giampietro Sandri, Diana Luise, Massimo Canali, Paolo Bosi

**Affiliations:** ^1^Department of Agricultural and Food Sciences, University of Bologna, Bologna, Italy; ^2^Società Agricola La Pellegrina s.p.a., Verona, Italy

**Keywords:** health, antimicrobial use, environment, economic impact, pig

## Abstract

The aim of this case study was to quantify antibiotic (AB) use in Italian weaning (W) and fattening (F) units differentiated for porcine reproductive and respiratory syndrome (PRRS) occurrence. Farms were classified as either PRRS negative (–) or PRRS positive (+) based on the circulation of the virus among the animals. In all the farms, the modified live PRRS virus (PRRSV) vaccine was provided to all the animals. In the PRRS– farms, the level of circulating antibodies was low, and the disease, in its clinical form, did not occur. In the PRRS+ farms, the level of circulating antibodies against the virus was high, and the disease was recurrent. Data regarding AB consumption were collected from 2017 to 2020, and the active compounds (ACs) were expressed as milligrams of AC/total kilogram of body weight (BW) produced. Each AC was classified into one of four categories according to the European Medicines Agency classification of ABs for prudent and responsible use in animals: Avoid, Restrict, Caution, and Prudence. Data regarding the ACs in each category were analyzed using a linear model that included production phase, PRRS status, and their interaction as factors. Performance parameters, average age of the pigs at the end of each phase, daily live weight gain, feed-to-gain ratio, total losses, cost index, and medication costs were significantly influenced by the PRRS chain. The use of class B ABs was not affected by production phase or PRRS status. Conversely, for class C ABs, interaction between the two factors (*p* = 0.02) was observed; W/PRRS+ and F/PRRS+ showed the greatest AB use for this class (*p* = 0.003). For class D ABs, the interaction was significant (*p* = 0.01); class C and D ABs were used more in the weaning (*p* = 0.07) than in the fattening phase (*p* = 0.003). For the weaning phase, the use of class C and D ABs was greater in the PRRS+ than in the PRRS– chain (*p* < 0.01). In conclusion, PRRS status affected the growth of pigs and economic performance. Moreover, PRRS status significantly influenced the use of ABs during all the growing periods with the greatest impact being on the weaning phase.

## Introduction

A new porcine reproductive and respiratory syndrome (PRRS) was first recognized in 1987; however, the causative virus (PRRSV) was first isolated in the Netherlands in 1991 and soon thereafter in the USA (1, 2). Since the discovery of this virus, much has been learned about it and its related diseases. A modified-live vaccine became available for pigs and has been widely used in all pig-producing areas. The widespread and appropriate use of vaccines has reduced the morbidity and mortality of pigs (3, 4). However, PRRS is still one of the most prevalent swine diseases, together with porcine influenza, and has a huge economic impact worldwide. The virus and the syndrome continue to evolve with clinical variations of the disease (3), making it difficult to find an effective vaccine-based prevention strategy.

Several aspects of PRRSV evolution and its interaction with the host are still poorly understood and are largely based on knowledge learned from *in vitro* or *in vivo* experimental infections (5). However, it is known that PRRSV has a tropism for macrophages of lymphoid tissues and lungs in which it mainly replicates (6–8).

Infection by PRRSV is not the only cause of death in nursery pigs; the secondary infections can often also be the cause of death; PRRSVs are frequently coinfecting with the other viruses or bacteria, which are most commonly found on pig farms (9–12). In fact, some published papers argue that an antibiotic-free production strategy could be risky in a PRRSV-endemic setting, especially if other bacterial coinfections are involved since pigs are exposed to severe clinical disease (13). Obviously, the judicious use of ABs can improve animal health (13). Furthermore, there are some ABs that may have antiviral effects. For instance, it seems that several quinolone-containing ABs inhibit the replication of PRRSV (14, 15).

Unfortunately, the increased and inappropriate use of some ABs to control bacterial infections in veterinary clinics have increased the risk of occurrence and diffusion of multidrug-resistant bacteria (12). Antibiotic resistance is one of the top health issues of major international health organizations. Among the various uses of ABs, low-dose, prolonged courses of ABs in food-producing animals or their use in nonbacterial diseases create ideal selective conditions for the propagation of resistant strains (16). Although antimicrobials are necessary for human and animal health, two thirds of future worldwide growth of the use of antimicrobials is predicted to be in animal production, particularly for the pig chain. According to the European Medicines Agency (EMA) Report, from 2010 to 2018, the estimated weight at AB treatment of livestock and slaughtered animals decreased more than 10% in Italy, unlike other countries in which it remained relatively stable or increased. Nevertheless, Italy is one of the countries in Europe in which the sales of veterinary antimicrobial agents for food-producing animals is the highest. Moreover, it is most commonly used in premixed or oral solutions while the use of injectable, intramammary, or intrauterine preparations is very low (17).

For these reasons, greater monitoring AB use is needed, highlighting risk factors in the various production phases and assessing whether the implemented control strategies are working or not. It should be noted that, in Italy, the pig supply chain is very fragmented; therefore, it is not easy to collect data regarding the use of medicine at different production stages.

The aim of this study was to investigate how the serological status (seropositive or seronegative) related to PRRS affected production performances and AB use in Italian weaning and fattening units. The authors' hypothesis was that a PRRS seronegative status positively influenced production performance, with animals being more productive and resistant to environmental bacterial infection, which allowed lower AB consumption.

## Materials and Methods

Data related to AB consumption and production performance were collected from 2017 to 2020. All the farms included in the study bred the Italian “heavy” pig, namely approved Large White, Landrace, and Duroc genetic lines and crossbreeds, listed in the production guidelines for Parma and San Daniele PDO cured ham; the pigs are typically slaughtered at ~160–170 kg of body weight (18). For this production, pigs are generally reared sequentially on two different farms, one for the nursery phase (approximately up to 70 days of age—“weaning phase”) and one for the growing-finishing phase (“fattening phase”) (18). For this study, each farm was classified as either PRRS negative (–) or PRRS positive (+) based on the circulation of the virus among the animals. The PRRS status of each single pig-flow was based on the combined use of both PCR (from blood, processing fluids, oral fluids, tissues) and serology (IDEXX–PRRS Elisa). On PRRS– farms, in the farrowing unit, all sows and gilts were still vaccinated, with both modified live vaccine and killed vaccine, administered intramuscularly, as a precautionary measure adopted after the first clinical outbreak of PRRS (2008–2009). In addition to this, PRRS serology (over 170 groups/2,500 pigs) was routinely carried out at the end of the nursery period on each group, and each group of pigs has constantly been negative since at least 2015–2016. On PRRS+ farms, all the animals were vaccinated; however, the level of circulating antibodies against the virus was markedly increased as compared with the PRRS– farms and the disease, in its clinical form, was recurrent. All the farms belonged to a fully integrated system and were organized into a multi-site production system in which pigs of the same farrowing unit were reared in predetermined weaning and fattening units year by year to avoid cross-contamination between the production chains.

Data were collected during the weaning and the fattening production phase; 115,970 pigs from PRRS– farms and 65,331 from PRRS+ farms were included in the weaning phase, and 108.248 pigs from PRRS– farms and 54,410 from PRRS+ farms were included in the fattening phase.

The growth performance indicators taken into account for both the weaning and the fattening phases were the number of pigs at the end of each phase, age, weight at the beginning and the end of each phase, and feed intake. The body weight produced (BW) was assessed from these data as was the average daily gain (ADG) and the feed to gain (F:G) ratio.

Moreover, the following parameters were recorded for each production cycle: total losses, medication costs, and efficiency index. The total losses were expressed as the total number of dead animals during the phase taken into account. Medication costs were calculated as the cost of each medical product divided by the kilograms of meat produced for each phase. The cost index, or efficiency index, included all the costs related to piglets, dead animals (total losses), veterinary medication, and the total cost of the feed used. Since the ingredients used for the feed are subject to strong price variations, the cost of the feed was maintained constant to avoid distortion in the index and to allow comparison between the years. For each cycle, the AB use was expressed as milligrams of active compound (AC) per kilogram of body weight (BW) produced. Each AC was classified according to the EMA classification of ABs for prudent and responsible use in animals. The classification ranks ABs by considering both the risk that their use in animals causes to public health through the possible development of antimicrobial resistance and the need to use them in veterinary medicine. It has four categories, from A to D: Avoid, Restrict, Caution, and Prudence, respectively. The “Restrict” category includes ABs that are critically important for human medicine, and their use in animals should be restricted to mitigate the risk to public health. For this reason, class B ABs should be used only when there are no effective alternatives in class C or D. Category C (“Caution”) covers ABs for which few alternatives are available in certain veterinary indications. These ABs should only be used when there are no antimicrobial substances in category D that would be clinically effective.

Category D (“Prudence”) includes ABs that should be used as first-line treatment, whenever possible. These ABs can be used in animals in a prudent manner (19). The AB use for each class was calculated as the sum of all the ABs (mg) administered that belonged to the same class. For each AB administered, the amount of BW treated was also calculated. This was one way to disregard the different dosages, according to the drug. However, the absolute values change according to the size of the supply chain and annual production. Thus, the data were presented in relative terms as values for each AB in the class over the sum of all the quantities of BW treated of the class, and were visualized by constructing graph rings. It is clear that, in some cases, the same animals may have been treated with multiple drugs in the same phase; therefore, the quantities add up.

### Statistical Analysis

The data of pig performance and AB consumption for each class and for the main individual ABs were considered to be arranged in a 2 × 2 factorial design with the following factors: 1) the two rearing phases (weaning and fattening) and 2) the classification of the production chain of the farms (positive or negative for PRRS). The data were thus analyzed using analysis of variance (by Proc GLM of SAS Inst., Inc., Cary, NC), considering these two main factors, their interaction and the effect of the year of rearing (4 years: 2017, 2018, 2019, 2020). Thus, the experimental unit was the observation in a given year on a farm specialized for a given phase, classified as negative or positive for PRRS. When the interaction was statistically significant, the differences between the means were tested using the Tukey test.

## Results

### Pig Performance

The effects of the parameters tested on the performance of weaning and fattening pigs are summarized in Table 1. There were no differences regarding pig performance over the years, except for the F:G ratio (*p* = 0.01), which decreased from 2017 (2.76) to 2020 (2.5). The production chain did not affect the starting live weight and the final live weight. The average age of the pigs at the end of each phase tended to be greater in the PRRS+ than in the PRRS– production chain (*p* = 0.058). The daily live weight gain (DLWG) was lower in the PRRS+ than in the PRRS– chain (*p* = 0.003) as the F:G ratio, total losses (TLs), the cost index (CI), and medication costs (MCs), which were significantly greater in the PRRS+ than in the PRRS– chain (*p* = 0.009, *p* = 0.001, *p* = 0.008, and *p* = 0.002, respectively). As expected, the parameters related to growth performance were significantly affected by the production phase (*p* < 0.01). The production phase also had an impact on the CI and the MCs, which both increased in the weaning period (*p* < 0.0001 and *p* = 0.002, respectively). Nevertheless, the production phase did not affect the TLs, expressed as whole mortality. Finally, statistically significant interactions between the production chain and phase were observed for TLs (*p* = 0.04), the CI (*p* = 0.04), and MCs (*p* = 0.005). In detail, these parameters were markedly increased in the weaning phase in the PRRS+ as compared with the PRRS– chain while no effect was detected in the fattening phase. In PRRS+, MCs were higher in the weaning phase compared with the fattening phase, but this was not the case in the PRRS– chain.

**Table 1:** General production data in the pig production chains differentiated by the occurrence of porcine reproductive and respiratory syndrome.

**Rearing phase:**	**Weaning**		**Fattening**		**SEM**	**Year**				**SEM**	***P*** **-value**			
**Productive chain^1^:**	**PRRS–**	**PRRS+**	**PRRS–**	**PRRS+**		**2017**	**2018**	**2019**	**2020**		**Chain**	**Phase**	**C × P**	**Year**
Pig (thousand)	116.0	65.3	108.2	54.4	6.1	84.1	79.8	81.9	98.1	6.14	<0.0001	0.16	0.80	0.21
Starting live weight, kg	7.1	6.9	32.0	31.7	0.8	18.4	18.8	21.1	19.3	0.8	0.75	<0.0001	0.92	0.12
Final live weight, kg	32.2	31.3	170.9	168.5	1.1	99.2	100.5	101.5	101.7	1.1	0.16	<0.0001	0.50	0.42
Average age, days	55.9	59.8	199.0	203.4	1.9	132.1	130.7	126.4	128.9	1.9	0.058	<0.0001	0.90	0.25
Daily live weight gain, g	449	407	699	674	9.0	541	559	566	563	8.57	0.003	<0.0001	0.36	0.23
Feed to gain	1.71	1.83	3.43	3.62	0.05	2.76	2.72	2.6	2.5	0.05	0.009	<0.0001	0.51	0.01
Total losses^2^	2.55^A^	14.49^B^	4.64^A^	9.04^AB^	1.61	9.47	9.72	5.92	5.61	1.61	0.001	0.32	0.04	0.20
Cost index^2^	1.63^aB^	1.91^bB^	0.88^A^	0.93^A^	0.05	1.41	1.37	1.27	1.3	0.05	0.008	<0.0001	0.04	0.20
Medication costs^2^, €/kg	0.04^A^	0.12^B^	0.03^A^	0.04^A^	0.01	0.07	0.06	0.04	0.04	0.01	0.002	0.002	0.005	0.33

1 *PRRS–, the production chain originated from sows seronegative for porcine reproductive and respiratory syndrome; PRRS+, the production chain originated from sows seropositive for porcine reproductive and respiratory syndrome*.

2 *Means with differently labeled letters are significantly different at p <0.05 (lowercase) or p <0.01 (uppercase)*.

### Antibiotic Consumption Divided by Class

Table 2 reports the consumption of ABs in the pig production chains differentiated by the occurrence of PRRS, expressed as milligrams of AC per total kilogram of meat produced (mg/kg). The consumption of class B ABs was relatively low in both the weaning and the fattening phases, and within PRRS negative and positive production chains. Furthermore, for this AB class, no difference was observed between the factors tested.

**Table 2:** Consumption of antibiotics in the pig production chain differentiated by the occurrence of porcine reproductive and respiratory syndrome (expressed as mg of active compound per total kg of meat produced).

**Rearing phase:**	**Weaning**		**Fattening**		**SEM**	**Year**				**SEM**	***P*** **-value**			
**Productive chain^1^:**	**PRRS–**	**PRRS+**	**PRRS–**	**PRRS+**		**2017**	**2018**	**2019**	**2020**		**Chain**	**Phase**	**C × P**	**Year**
**Class of antibiotic—B^2^**														
	0.85	0.86	0.05	0.22	0.38	0.42	0.59	0.51	0.46	0.37	0.82	0.11	0.84	0.99
**Class of antibiotic—C^2,3^**														
	21.7^A^	134.5^b*B*^	32.4^A^	54.5^a^	16.5	73.7	70.5	34.9	64.1	16.5	0.003	0.07	0.02	0.38
**Class of antibiotic—D** **^2,3^**														
	164^A^	782^B^	57^A^	81^A^	98.0	393	342	182	167	98	0.097	0.003	0.01	0.32

1 *PRRS–, the production chain originated from sows seronegative for porcine reproductive and respiratory syndrome; PRRS+, the production chain originated from sows seropositive for porcine reproductive and respiratory syndrome*.

2 *Classification of antibiotics in the European Union—B, Restrict; C, Caution; D, Prudence*.

3 *Means with differently labeled letters are significantly different at p < 0.05 (lowercase) or p < 0.01 (uppercase)*.

As concerns the other two classes of ABs, the interaction between production chain and phase was statistically significant for class C (*p* = 0.02) and class D (*p* = 0.01). For the weaning phase, the use of ABs of classes C and D was greater in the PRRS+ than in the PRRS– chain (*p* < 0.01) with an increase of 520% and of 377%, respectively. Conversely, no difference was observed in the fattening phase. In the PRRS+ chain, class C (*p* < 0.05) and class D (*p* < 0.01) AB use was greater in the weaning as compared with the fattening phase, although the same difference was not detected for the PRRS– farms.

No significant differences were observed over the years for all the AB classes.

### Use of Active Compounds

Within class C ABs, florfenicol and lincomycin were the most used active compounds in both the weaning and the fattening phases. Florfenicol accounted for 24.0 and 24.2% of class C ABs in the weaning and the fattening phases, respectively. Lincomycin accounted for 22.7 and 32.5% of class C ABs in the weaning and the fattening phases, respectively. Regarding class D, the most used active compounds in the weaning phase were amoxicillin (average 38.1%) and spectinomycin (average 29.5%) while, in the fattening phase, they were ampicillin (average 21.9%) and dicloxacillin (average 21.5%).

**Figure 1:**
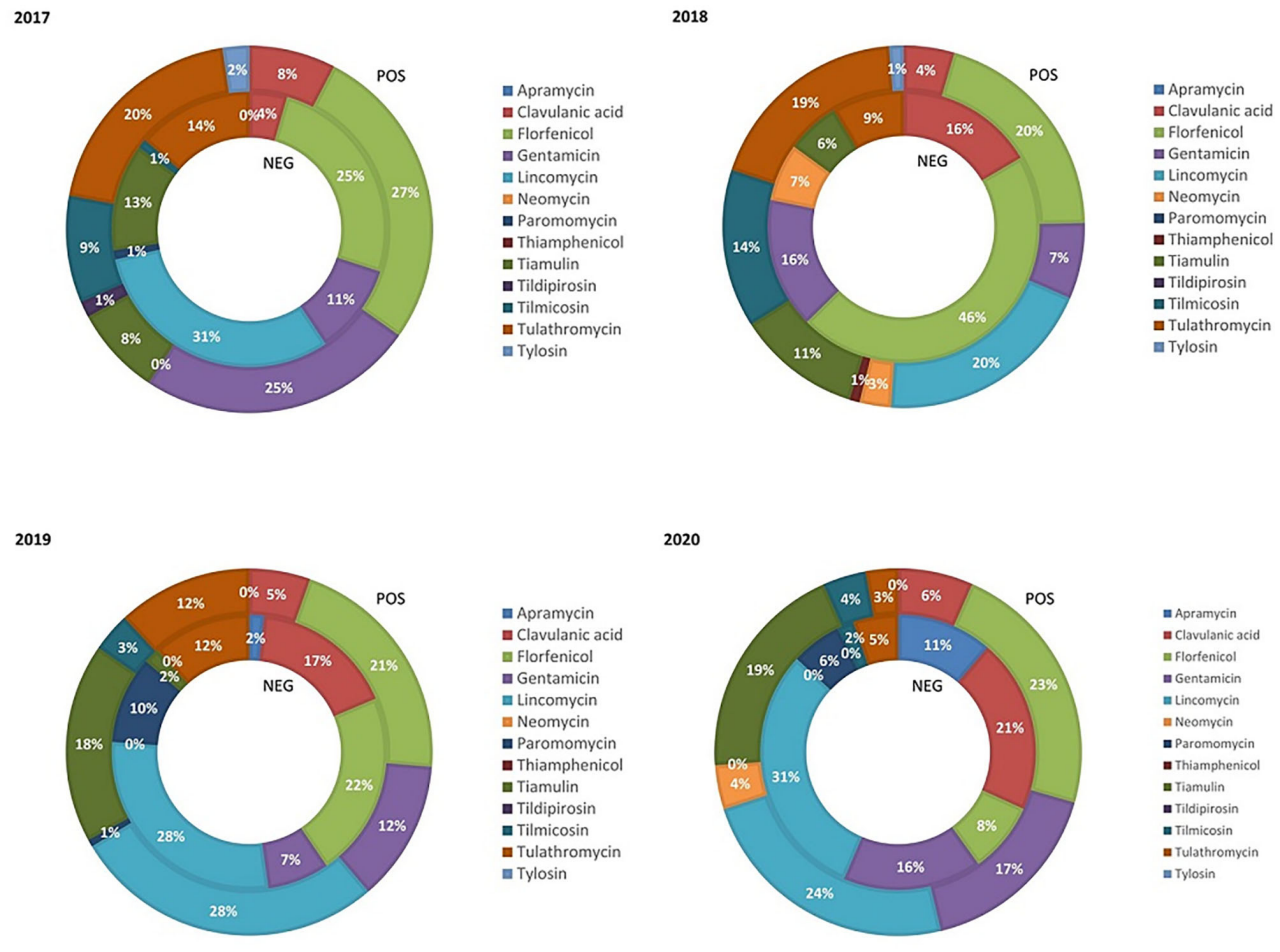
Relative quantity of weaning pig weight treated with class C antibiotics in the PRRS negative (NEG) and PRRS positive (POS) production chains from 2017 to 2020.

The relative BW treated with class C ABs during the weaning phase in PRRS+ and PRRS– chains from 2017 to 2020 is reported in Figure 1. The use of tulathromycin decreased within both production chains from 2017 to 2020. The relative BW treated with clavulanic acid remained quite constant in the PRRS+ chain (average 5.75%) and significantly increased in PRRS– chain from 2017 (4%) to 2020 (21%). Similarly, the use of florfenicol was constant in the PRRS+ chain (average, 22.75%), but it was variable in the PRRS– chain, reaching a maximum in 2018 (46%). The use on lincomycin decreased within the PRRS– chain from 31% in 2017 to 0% in 2018%; however, it then increased again in 2019 and 2020 by 28 and 31%, respectively. In the PRRS+ chain, lincomycin ranged from 20 to 28%, depending on the year. Gentamicin use remained relatively constant (average 12.5%) in the PRRS– chain over time while it decreased in the PRRS+ chain from 2017 (25%) to 2018 (7%) and then increased until 2020 (17%). The relative BW treated with tiamulin and tilmicosin varied little. Finally, the use of apramycin, thiamphenicol, tildipirosin, and tylosin was low or even absent.

**Figure 2:**
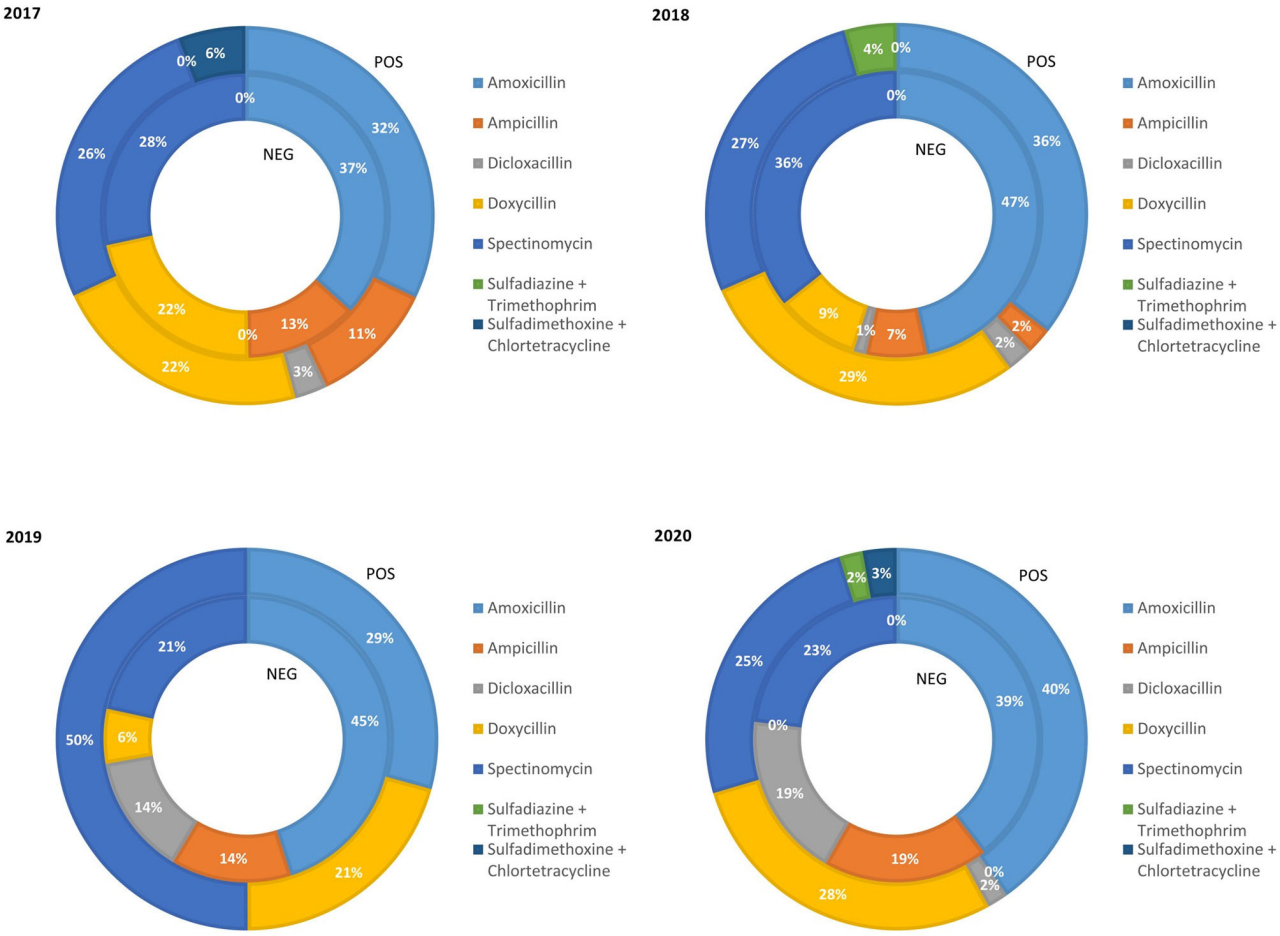
Relative quantity of weaning pig weight treated with class D antibiotics in the PRRS negative (NEG) and PRRS positive (POS) production chains from 2017 to 2020.

The relative amount of weaned pig weight treated with class D ABs in the PRRS+ and the PRRS– production chains from 2017 to 2020 is reported in Figure 2. The percentage of pigs treated with amoxicillin in the PRRS+ and the PRRS– chains during the period considered was quite high (average 34.25 and 43%, respectively, over the 4 years). The percentage of pigs treated with ampicillin decreased in the PRRS+ chain from 11% in 2017 to 2% in 2018 and was then zero until 2020 while, in the PRRS– chain, it decreased from 2017 (13%) to 2018 (7%) and then increased until 2020 (19%). Instead, the percentage of pig weight treated with dicloxacillin in the PRRS– chain increased from 0% in 2017 to 19% in 2020 while, in the PRRS+ chain, it remained low and constant over the years (average 1.75%). The percentage of pig weight treated with doxycycline gradually decreased in the PRRS– chain from 22% in 2017 to 0% in 2020, and remained quite constant in the PRRS+ chain (average 25%). The percentage of pig weight treated with spectinomycin was quite constant in the PRRS– chain (average 27%) while, in the PRRS+ chain, it increased from 27% in 2018 to 50% in 2019 and then decreased again to 25% in 2020. The percentage of pig weight treated with sulfadiazine + trimethoprim and sulfadimethoxine + chlortetracycline remained constant and limited over the years.

**Figure 3:**
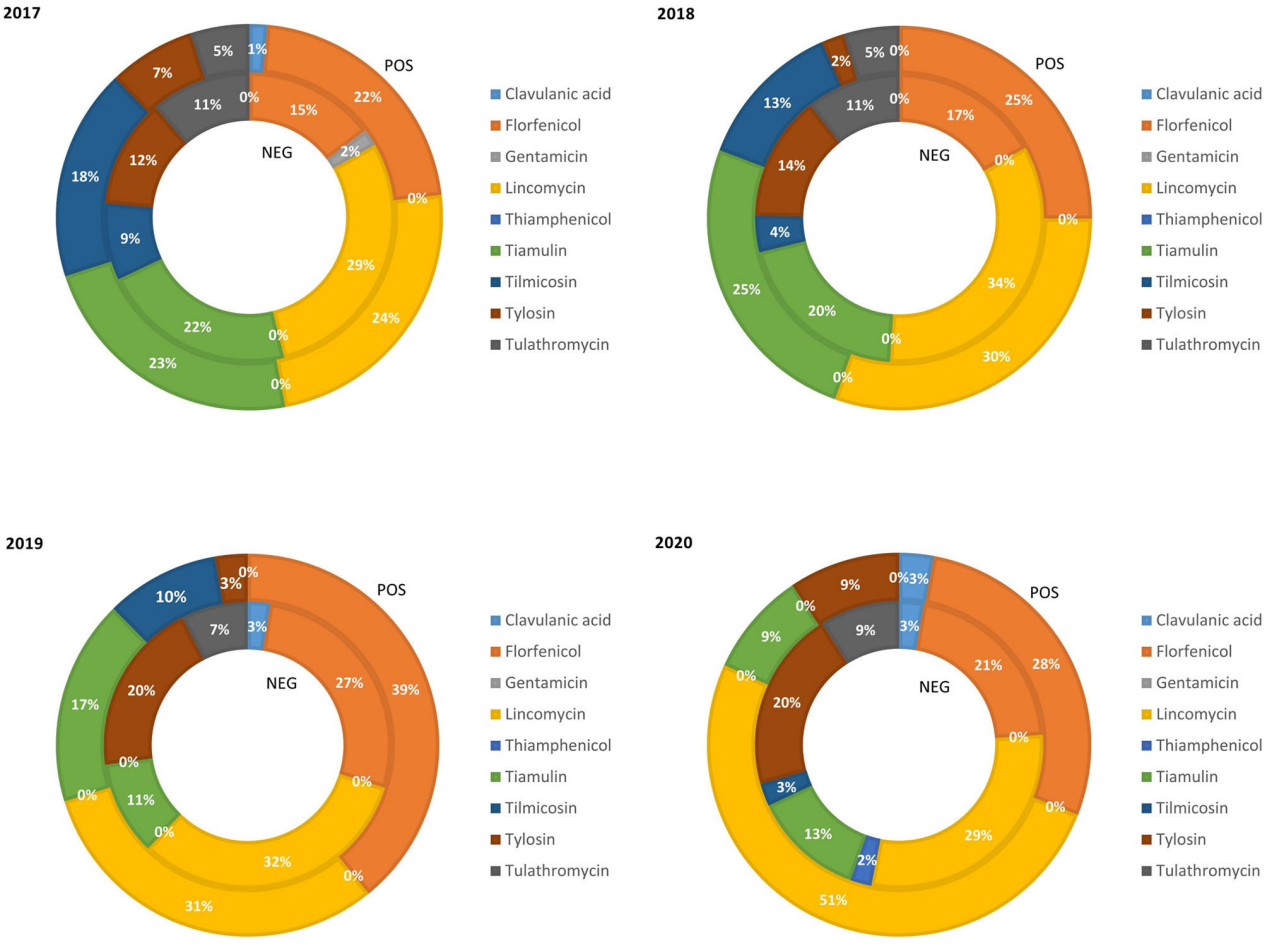
Relative quantity of fattening pig weight treated with class C antibiotics in the PRRS negative (NEG) and PRRS positive (POS) production chains from 2017 to 2020.

The relative quantity of fattening pig weight treated with class C ABs in the PRRS+ and PRRS– chains from 2017 to 2020 is reported in Figure 3. In this rearing phase, the rate of pigs treated with clavulanic acid, gentamicin, and thiamphenicol remained very limited (average 1.25, 0.25, and 0.25%, respectively). The percentage of fattening pig weight treated with florfenicol in the PRRS– chain increased from 2017 (15%) to 2019 (27%) and then decreased again in 2020 (21%). Similarly, in the PRRS+ chain, it significantly increased from 2017 (22%) to 2019 (39%) and then decreased in 2020 (28%). The percentage of pig weight treated with lincomycin remained quite constant in the PRRS– chain (average 31%) while in the PRRS+ chain, it increased significantly from 24% in 2017 to 51% in 2020. The percentage of pigs treated with tiamulin in the PRRS– chain decreased from 2017 (22%) to 2019 (11%) and then remained stable until 2020 (13%); similarly, in the PRRS+ chain, it decreased from 2017 (23%) to 2020 (9%). The percentage of tilmicosin decreased constantly in the PRRS+ and PRRS– chains from 2017 (18 and 9%, respectively) to 2020 until it was zero. The percentage of pigs weight treated with tylosin in the PRRS+ chain decreased from 7% in 2017 to 2% in 2018 and then increased until 2020 (9%), while, in the PRRS– chain, it increased constantly from 2017 (12%) to 2018 (20%) and then remained constant. The percentage of tulathromycin in the PRRS+ chain was constant in 2017 and 2018 (5%) and then became zero from 2019 while, in the PRRS– chain, it remained quite constant over the years (average 9.5%).

**Figure 4:**
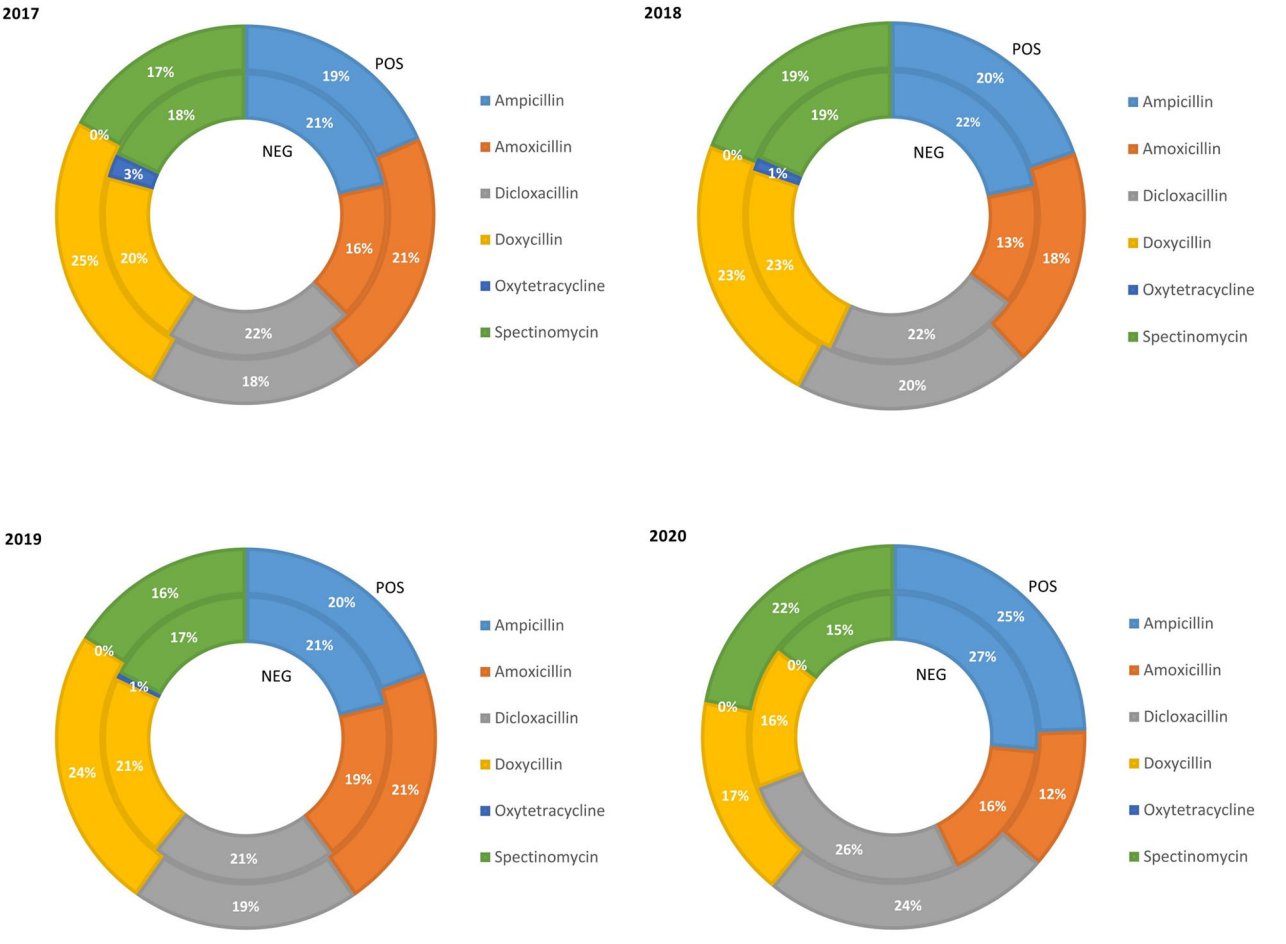
Relative quantity of fattening pig weight treated with class D antibiotics in the PRRS negative (NEG) and PRRS positive (POS) production chains from 2017 to 2020.

The relative quantity of fattening pigs treated with class D ABs in the PRRS positive and negative chains from 2017 to 2020 is reported in Figure 4. The percentage of pigs treated with ampicillin was similar and slightly increased in both chains (from 20% in 2017 to 25% in 2020). Amoxicillin remained relatively constant in the PRRS– chain over the years (average 16%) while, in the PRRS+ chain, it remained stable until 2019 and decreased in 2020 (12%). The percentage of dicloxacillin use did not change from 2017 to 2019 in either chain, with an average of 21.6% in the PRRS– chain and 19% in the PRRS+ chain, and slightly increased in 2020 (26 and 24%, respectively). On the contrary, the percentage of pigs weight treated with doxycycline was constant in both the PRRS– (average 21.3%) and the PRRS+ (average 24%) chains between 2017 and 2019 and then decreased in 2020 (16 and 17%, respectively). Moreover, the percentage of pig weight treated with spectinomycin remained relatively stable in the PRRS– chain (average 17.25%), however slightly decreasing in 2020 (15%), and also in the PRRS+ chain (average 18.5%), increasing in 2020 (22%). Oxytetracycline remained very low in the PRRS– chain, decreasing from 3% to zero between 2017 and 2020 while, in the PRRS+ chain, it was always zero.

The effects of the parameters tested on the use of specific ACs is reported in Table 3. These ACs were selected because of their widespread use in the production chains. The production phase only marginally influenced the use of amoxicillin (*p* = 0.08), the use of which was higher in the weaning phase than in the fattening phase. Interaction between production chain and phase (*p* = 0.02) was observed for the use of florfenicol. In the weaning phase, the use of florfenicol was higher in the PRRS+ than in the PRRS– chain (*p* < 0.01); conversely, no difference was seen in the fattening phase. In the PRRS+ chain, its use was higher in the weaning phase (*p* < 0.01) than in the fattening phase; however, this was not seen in the PRRS– chain. The production phase marginally influenced the use of lincomycin (*p* = 0.10) with high use of this active compound in the PRRS+ than in the PRRS– production chain. Spectinomycin was used more in the weaning phase than in the fattening phase (*p* < 0.0001), whatever the production chain was.

**Table 3:** Consumption of two main class C antibiotics, florfenicol and lincomycin, and two main class D antibiotics, amoxicillin and spectinomycin, in the pig production chain differentiated by the occurrence of porcine reproductive and respiratory syndrome (expressed as mg of active compound per total kg of meat produced).

**Rearing phase:**	**Weaning**		**Fattening**		**SEM**	**Year**				**SEM**	***P*** **-value**			
**Productive chain^1^:**	**PRRS–**	**PRRS+**	**PRRS–**	**PRRS+**		**2017**	**2018**	**2019**	**2020**		**Chain**	**Phase**	**C × P**	**Year**
Florfenicol	2.2^A^	32.8^B^	1.0^A^	4.1^A^	4.7	17.1	13.1	4.81	5.1	4.7	0.006	0.01	0.02	0.25
Lincomycin	7.4	30.5	5.8	24.8	10.9	11.1	11.8	10.6	35	10.9	0.10	0.75	0.86	0.33
Amoxicillin	8.0	32.9	0.7	1.7	10.9	15.6	13.3	8.01	6.4	10.9	0.24	0.08	0.28	0.93
Spectinomycin	16.5	19.7	0.4	1.9	2.1	11.5	12.1	6.0	8.9	2.1	0.31	<0.0001	0.71	0.22

1 *PRRS–, the production chain originated from sows seronegative for porcine reproductive and respiratory syndrome; PRRS+, the production chain originated from sows seropositive for porcine reproductive and respiratory syndrome*.

1 *Means with differently labeled letters are significantly different at p < 0.01*.

No significant differences were observed during the time of the survey for all four ABs.

## Discussion

The PRRS is one of the most important viral-based illnesses, having a severe impact on modern pig production. The results of this survey clearly showed the link between the active circulation of the PRRSV and the use of antimicrobials in weaning and fattening units. Even if the greatest losses due to PRRS are shown in weaners and growers, there are some studies regarding PRRS that are focused on the detrimental effect of this syndrome in breeding units in terms of performance (20) and economic losses (21, 22). However, the present survey pointed out that a positive PRRS status negatively influences pig growth performance in both weaning and fattening units, although, in the latter case, the impact was less evident. These data confirmed the observation of Schweer et al. (23) who reported a negative effect on ADG and the F:G ratio in growing pigs infected with PRRSV due to the reduction of the dry matter digestibility and protein accretion rates. Moreover, the effect of the infection on the additional energy expenditure to sustain the immune system activation was not negligible. In fact, the reallocation of amino acid to the production of energy, as well as supporting the syntheses of the components of the immune system, reduced the activation of the protein synthesis pathways, causing a reduction in skeletal muscle growth (24). It also seemed that a reduction in feed intake, mediated by the cytokine release, may have indirectly induced myostatin expression, which inhibited muscle growth (25).

The same effect was seen even on farms infected with PRRSV in which vaccinated pigs had improved growth performance as compared with untreated pigs, in particular regarding body weight, ADG and the feed conversion ratio (FCR), and presented reduced morbidity and mortality and a reduced incidence of respiratory bacterial infections (26). In addition, the data also showed higher mortality on PRRS+ farms than on PRRS– farms. This also partially justified an increase in production cost since fixed production costs are distributed over a lower number of pigs produced (21, 27).

The present study demonstrated that class B AB use was the lowest during the considered period. Moreover, the present results agreed with other studies (28, 29) that identified penicillin and tetracycline as the most used families of ABs in Europe. Both penicillin and tetracycline belong to class D of the EMA classification, which includes ABs that should be used as first-line treatment, whenever possible.

Even if this study demonstrated that the amount of class B ABs did not decrease significantly during the period of time taken into account, as sustained by Tarakdjian et al. (30), there are active compounds, the use of which was significantly reduced in the past. The use of colistin, for instance, which belongs to class B Abs, together with other polymyxins (27, 31), was markedly reduced over this period of time. Colistin is considered to be a critically important AC in human medicine since it represents one of the few available treatments against multidrug-resistant bacterial infection. However, colistin has been used against *Enterobacteriaceae* for many years, particularly in pigs, as one of the most effective ABs for treating weaning diarrhea caused by enterotoxigenic *Escherichia coli* (31, 32).

Both the suckling and the Post-weaning periods represent critical phases in pig production, having the highest risk of disease. After farrowing and weaning, piglets are particularly vulnerable to infections caused by pathogens. For these reasons, during the suckling and the Post-weaning periods, the use of ABs is typically increased in pig production (28, 33). Treatment with penicillin or aminopenicillin, as well as with amoxicillin or ampicillin, which belong to class D ABs, can usually also be effective in this phase (34, 35). The present study demonstrated greater use of these ABs in the weaning as compared with the fattening period, especially in PRRS+ farms. As expected, the consumption of class C ABs was significantly greater in the PRRS+ chain. This class of Abs contains ACs that represent the first-choice treatment for respiratory or enteric diseases caused by *Actinobacillus pleuropneumoniae, Pasteurella* spp., *Streptococcus* spp., *Clostridium* spp., *E.coli*, or *Salmonella* spp. Coinfections are frequent on pig farms; in particular, animals infected with PRRSV are more susceptible to other diseases. The Porcine Respiratory Disease Complex describes coinfections of PRRSV-like viruses and bacteria, such as *Actinobacillus pleuropneumoniae, Mycoplasma hyopneumoniae*, and *Bordetella bronchiseptica*. The PRRSV is frequently also isolated with *Steptococcus suis, Staphylococcus, Haemophilus parasuis, Pasteurella, Salmonella, Proteus*, and *Morganella* (11, 12, 36, 37).

In this study, the chain and production phase did not significantly influence the use of amoxicillin. In fact, amoxicillin is used on a large scale in pig production, also in association with clavulanic acid, because it is effective against several systemic, respiratory, and enteric infections (38), even if some argue that there are bacteria that are also becoming resistant to this AB (12).

This survey demonstrated that florfenicol was widely used in the weaning period, more in the PRRS+ chain rather than in the PRRS– chain. This was probably due to its widespread activity against the pathogens that are commonly secondary to PRRS syndrome. Florfenicol has antibacterial activity against major swine respiratory pathogens (*Pasteurella multocida, Haemophilus sommus, Actinobacillus pleuropneumoniae*) and many other Gram+ and Gram– bacteria (*E. coli, Salmonella typhimurium, Streptococcus suis, Staphylococcus aureus*). Its antibacterial activity is strengthened when used in combination with tilmicosin or doxycycline hydrochloride, such as the combination of two or more ABs having active interaction is an important possibility for overcoming the problem of secondary infections (39–41), even if Holmer et al. (42) pointed out the increase in resistance of *E. coli* to florfenicol.

Lincomycin was also widely used in this survey, without big differences between chains and phases. Lincomycin is commonly used on pig farms for the treatment of gastrointestinal infections, such as ileitis caused by *Lawsonia intracellularis* (43, 44). In addition, it seems that some pathogens, such as *Haemophilus parasuis* and *Pasteurella multocida*, are resistant to many ABs but not to lincomycin and quinolone whereas *Streptococcus suis* is also resistant to lincomycin and many other ACs (45).

The European Surveillance of Veterinary Antimicrobial Consumption (ESVAC) report (17) showed that the most used ABs for food-producing animals in Italy in 2018 were penicillin and tetracyclines while the use of aminoglycosides was relatively low. These data are in agreement with other studies that assessed antimicrobial use on European and Italian pig farms until 2017. These studies identified penicillin and tetracyclines as the most used ABs, likely due to their cost-effectiveness as compared with other ACs (28, 30).

This study demonstrated a slight reduction in the use of macrolides, such as tulathromycin and tilmicosin, but not of tilosin, in both phases. Most likely, the decreased use of this family of ABs could be attributed to the fact that they were considered to be critically important in human medicine (46). According to the ESVAC report, the sale of macrolides in Italy for food-producing animals decreased significantly from 2010 to 2018, even if it was still high as compared with the majority of other countries (17). The main reason that led to considering macrolides as highest priority critically important ABs (HPCIAs) was human campylobacteriosis. Due to increased resistance to fluoroquinolone, macrolides remain the only choice for treating this disease in humans. It should be emphasized that pig farming is a very minor source of *Campylobacter* spp. spread, unlike other production chains, such as poultry (47, 48).

The authors consider the data regarding the use of different classes of ABs (EMA classification) in the weaning and fattening units very interesting. It provides indications regarding AB use in Italy as well as on the impact of the PRRS on the use of the different classes of ABs. These data are unique for Italy in that they cover both a long period of time and a large number of animals, and are based on the actual kilograms of meat produced per year.

The PRRSV is one of the most important pathogens that has negatively influenced the global pig industry for a long time. An antiviral treatment has not yet been created for food-producing animals, probably owing to economic reasons. The eradication of this disease would allow avoiding huge economic losses and reducing the use of antimicrobials in the pig production systems, minimizing the development of antimicrobial resistance. There are novel strategies that seem to be promising other than the modified live vaccine; one of these attempts is to create a cross-protective chimeric virus vaccine and another attempts to restore the immune response of the host using interferon-inducible strains (49). There are currently vaccines available that reduce morbidity, mortality, treatment costs, and losses due to the PRRSV; however, they cannot completely prevent respiratory infection (26). Therefore, in addition to the use of vaccines, good biosecurity standards should always be applied, and animal welfare improved. Animals living with environmental enrichment, for instance, are associated with a reduction in manipulative oral behavior directed at pen mates and in stress, both of which lead to a reduced impact of infections (50, 51).

It is important to highlight that the majority of studies quantify the use of ABs at a population level, using the metric of the total mass (mg) of any AB active ingredient (or group) per population corrected unit (PCU). The latter considers the animal population and the estimated weight of the animals at the time of AB treatment (29). Commonly used concepts, such as UDD (used daily doses), ADD (animal daily dose), or DCD (defined course dose), were used to analyze the data. These parameters always correlated with the dosage of the AB and with the weight or the number of animals treated (52, 53). To quantify the overall use of antimicrobials per year, the defined daily dose/population correction unit (DDDvet/PCU), a method proposed by EMA is commonly used (30). In this study, the consumption of ABs was expressed in milligrams of AC per total kilogram of meat produced in a specific interval of time. This resulted in a more economic and practical appearance because it referred to the final product of one phase, thus to the net production. For practical purposes, to compare the use of different ABs or for grouping their use, calculation of the quantity of BW treated was also interesting since it did not take into account the pharmaceutical dose, although, to the authors' knowledge, this has never been proposed before.

## Conclusion

This study demonstrated that PRRSV infection affected growth performance and antimicrobial use in the Italian heavy pig production system. Circulation of the PRRSV within a herd negatively affected performance and led to a greater use of antimicrobials, especially for treating secondary infections. The present results demonstrated the low usage of class B ABs for both PRRS+ and PRRS– chains from 2017 to 2020. The use of class C and D ABs was greater when there was presence of disease. In the present study, class D Abs were the most used in the production systems.

The mode of using ACs changed during the 4 years of the study, even though a statistically significant overall reduction was not evidenced over time, probably due to the lack of data from individual farms. Finally, the data reported highlighted the fact that management strategies targeted to have stable herds negative for PRRS infection occurrence markedly improved the production performance and reduced the use of antimicrobials during the entire production cycle.

One limitation of the study was the impossibility of knowing the reasons for each treatment and of explaining all the variations observed regarding the ACs used over the years. Nevertheless, the present study was based on a large 4-year dataset; therefore, there was a large quantity of data that allowed providing, for the first time, a vision of the Italian reality regarding the use of ABs under well-defined conditions (PRRS) on a representative number of animals.

## Data Availability Statement

The datasets presented in this article are not readily available because the owner of the dataset is a company. Requests to access the datasets should be directed to PT, paolo.trevisi@unibo.it.

## Ethics Statement

Ethical review and approval was not required for the animal study because data were provided by a pig integratet group.

## Author Contributions

PT and PB designed the experiment. GS, LA, RR, and CR carried out the experiment and collected the data. PB analyzed the data. PT, LA, and PB conceptualized the article, compiled all the information, and prepared the article. MC, GS, and DL conceptualized the article, provided insights to the entire article, and contributed to the writing. All authors have read and approved the final article.

## Funding

The ROADMAP project (Rethinking of Antimicrobial Decision-systems in the Management of Animal Production) received funding from the European Union's Horizon 2020 research and innovation program under Grant Agreement No. 817626.

## Conflict of Interest

GS was employed by the company Società Agricola La Pellegrina s.p.a. The remaining authors declare that the research was conducted in the absence of any commercial or financial relationships that could be construed as a potential conflict of interest.

## Publisher's Note

All claims expressed in this article are solely those of the authors and do not necessarily represent those of their affiliated organizations, or those of the publisher, the editors and the reviewers. Any product that may be evaluated in this article, or claim that may be made by its manufacturer, is not guaranteed or endorsed by the publisher.

## References

[1] Terpstra C Wensvoort G Pol JMA. Experimental reproduction of porcine epidemic abortion and respiratory syndrome (mystery swine disease) by infection with Lelystad vims: Koch's postulates fulfilled. Vet Q. (1991) 13:131–6. 10.1080/01652176.1991.9694297 1949539

[2] Collins JE Benfield DA Christianson WT Harris L Hennings JC Shaw DP. Isolation of swine infertility and respiratory syndrome virus (Isolate ATCC VR-2332) in North America and experimental reproduction of the disease in gnotobiotic pigs. J VET Diagn Invest. (1992) 4:117–26. 10.1177/104063879200400201 1616975

[3] Lunney JK Benfield DA Rowland RRR. Porcine reproductive and respiratory syndrome virus: an update on an emerging and re-emerging viral disease of swine. Virus Res. (2010) 154:1–6. 10.1016/j.virusres.2010.10.009 20951175 PMC7172856

[4] Renukaradhya GJ Meng X-J Calvert JG Roof M Lager KM. Live porcine reproductive and respiratory syndrome virus vaccines: Current status and future direction. Vaccine. (2015) 33:4069–80. 10.1016/j.vaccine.2015.06.092 26148878

[5] Franzo G Dotto G Cecchinato M Pasotto D Martini M Drigo M. Phylodynamic analysis of porcine reproductive and respiratory syndrome virus (PRRSV) in Italy: Action of selective pressures and interactions between different clades. Infection, Genetics and Evolution. (2015) 31:149–57. 10.1016/j.meegid.2015.01.026 25660037

[6] Duan X Nauwynck HJ Pensaert MB. Virus quantification and identification of cellular targets in the lungs and lymphoid tissues of pigs at different time intervals after inoculation with porcine reproductive and respiratory syndrome virus (PRRSV). Vet Microbiol. (1997) 56:9–19. 10.1016/S0378-1135(96)01347-8 9228678

[7] Duan X Nauwynck HJ Pensaert MB. Effects of origin and state of differentiation and activation of monocytes/macrophages on their susceptibility to porcine reproductive and respiratory syndrome virus (PRRSV). Arch Virol. (1997) 142:2483–97. 10.1007/s007050050256 9672608 PMC7086874

[8] Beyer J Fichtner D Schirrmeir H Polster U Weiland E Wege H. Porcine Reproductive and Respiratory Syndrome Virus (PRRSV): kinetics of infection in lymphatic organs and lung. J Vet Med Series B. (2000) 47:9–25. 10.1046/j.1439-0450.2000.00305.x 10780169 PMC7183809

[9] Xu M Wang S Li L Lei L Liu Y Shi W. Secondary infection with Streptococcus suis serotype 7 increases the virulence of highly pathogenic porcine reproductive and respiratory syndrome virus in pigs. Virol J. (2010) 7:184. 10.1186/1743-422X-7-184 20696031 PMC2927530

[10] Jiang N Liu H Wang P Huang J Han H Wang Q. Illumina MiSeq sequencing investigation of microbiota in bronchoalveolar lavage fluid and cecum of the swine infected with PRRSV. Curr Microbiol. (2019) 76:222–30. 10.1007/s00284-018-1613-y 30554323

[11] Zhao G Zhang L Li C Zhao J Liu N Li Y. Identification of enterobacteria in viscera of pigs afflicted with porcine reproductive and respiratory syndrome and other viral co-infections. Microb Pathog. (2020) 147:104385. 10.1016/j.micpath.2020.104385 32659314 PMC7352111

[12] Xiangjin Yan Zeng J Li X Zhang Z Din AU Zhao K. High incidence and characteristic of PRRSV and resistant bacterial co-infection in pig farms. Microbial Pathog. (2020) 149:104536. 10.1016/j.micpath.2020.104536 32980472

[13] Dee S Guzman JE Hanson D Garbes N Morrison R Amodie D. A randomized controlled trial to evaluate performance of pigs raised in antibiotic-free or conventional production systems following challenge with porcine reproductive and respiratory syndrome virus. PLoS One. (2018) 13:e0208430. 10.1371/journal.pone.0208430 30521587 PMC6283559

[14] Cafruny WA Duman RG Rowland RR Nelson EA Wong GH. Antibiotic-mediated inhibition of porcine reproductive and respiratory syndrome virus (PRRSV) infection: a novel quinolone function which potentiates the antiviral cytokine response in MARC-145 cells and pig macrophages. Virology. (2008) 1:VRT.S527. 10.4137/VRT.S527

[15] Desmonts de Lamache D Moges R Siddiq A Allain T Feener TD Muench GP. Immuno-modulating properties of Tulathromycin in porcine monocyte-derived macrophages infected with porcine reproductive and respiratory syndrome virus. PLoS One. (2019) 14:e0221560. 10.1371/journal.pone.0221560 31442273 PMC6707645

[16] Marshall BM Levy SB. Food animals and antimicrobials: impacts on human health. Clin Microbiol Rev. (2011) 24:718–33. 10.1128/CMR.00002-11 21976606 PMC3194830

[17] EMA-ESVAC - 2020 - sales-veterinary-AM-2010-2018-tenth-esvac-report.pdf. EMA-ESVAC. Sales of Veterinary Antimicrobial Agents in 31 European Countries in 2018.Trends from 2010 to 2018. Tenth ESVAC report sales (2020).

[18] Consorzio del Prosciutto di Parma. Prosciutto di Parma (Parma ham) Protected Designation of Origin. (1992). Available online at: https://www.prosciuttodiparma.com/wp-content/uploads/2019/07/Parma_Ham_Specifications_Disciplinare_Consolidato_Nov_13.pdf

[19] EMA. Categorization antibiotics European union. Answer to the request from the European Commission for updating the scientific advice on the impact on public health and animal health of the use of antibiotics in animals. EMA (2019).

[20] Torrents D Miranda J Gauger PC Ramirez A Linhares D. Effect of PRRSV stability on productive parameters in breeding herds of a swine large integrated group in Spain. Porcine Health Manag. (2021) 7:21. 10.1186/s40813-021-00203-4 33637120 PMC7908702

[21] Nathues H Alarcon P Rushton J Jolie R Fiebig K Jimenez M. Cost of porcine reproductive and respiratory syndrome virus at individual farm level – An economic disease model. Prev Vet Med. (2017) 142:16–29. 10.1016/j.prevetmed.2017.04.006 28606362

[22] Renken C Nathues C Swam H Fiebig K Weiss C Eddicks M. Application of an economic calculator to determine the cost of porcine reproductive and respiratory syndrome at farm-level in 21 pig herds in Germany. Porc Health Manag. (2021) 7:3. 10.1186/s40813-020-00183-x 33397503 PMC7784293

[23] Schweer W Schwartz K Patience JF Karriker L Sparks C Weaver M. Porcine reproductive and respiratory syndrome virus reduces feed efficiency, digestibility, and lean tissue accretion in grow-finish pigs1. Transl Anim Sci. (2017) 1:480–8. 10.2527/tas2017.0054 32704671 PMC7204981

[24] Helm ET Curry SM de Mille CM Schweer WP Burrough ER Zuber EA. Impact of porcine reproductive and respiratory syndrome virus on muscle metabolism of growing pigs1. J Anim Sci. (2019) 97:3213–27. 10.1093/jas/skz168 31212312 PMC6667233

[25] Escobar J van Alstine WG Baker DH Johnson RW. Decreased protein accretion in pigs with viral and bacterial pneumonia is associated with increased myostatin expression in muscle. J Nutr. (2004) 134:3047–53. 10.1093/jn/134.11.3047 15514274

[26] Kritas SK Alexopoulos C Kyriakis CS Tzika E Kyriakis SC. Performance of fattening pigs in a farm infected with Both Porcine Reproductive and Respiratory Syndrome (PRRS) virus and porcine circovirus type 2 following sow and piglet vaccination with an attenuated PRRS vaccine. J Vet Med Series A. (2007) 54:287–91. 10.1111/j.1439-0442.2007.00932.x 17650147

[27] Neumann EJ Kliebenstein JB Johnson CD Mabry JW Bush EJ Seitzinger AH. Assessment of the economic impact of porcine reproductive and respiratory syndrome on swine production in the United States. J Am Vet Med Assoc. (2005) 227:385–92. 10.2460/javma.2005.227.385 16121604

[28] Lekagul A Tangcharoensathien V Yeung S. Patterns of antibiotic use in global pig production: a systematic review. Vet Anim Sci. (2019) 7:100058. 10.1016/j.vas.2019.100058 32734079 PMC7386699

[29] O'Neill L Rodrigues da Costa M Leonard FC Gibbons J Calderón Díaz JA McCutcheon G. Quantification, description and international comparison of antimicrobial use on Irish pig farms. Porc Health Manag. (2020) 6:30. 10.1186/s40813-020-00166-y 33062293 PMC7549222

[30] Tarakdjian J Capello K Pasqualin D Santini A Cunial G Scollo A. Antimicrobial use on italian pig farms and its relationship with husbandry practices. Animals. (2020) 10:417. 10.3390/ani10030417 32131557 PMC7143824

[31] Catry B Cavaleri M Baptiste K Grave K Grein K Holm A. Use of colistin-containing products within the European Union and European Economic Area (EU/EEA): development of resistance in animals and possible impact on human and animal health. Int J Antimicrob Agents. (2015) 46:297–306. 10.1016/j.ijantimicag.2015.06.005 26215780

[32] Rhouma M Fairbrother JM Beaudry F Letellier A. Post weaning diarrhea in pigs: risk factors and non-colistin-based control strategies. Acta Vet Scand. (2017) 59:31. 10.1186/s13028-017-0299-7 28526080 PMC5437690

[33] Sjölund M Postma M Collineau L Lösken S Backhans A Belloc C. Quantitative and qualitative antimicrobial usage patterns in farrow-to-finish pig herds in Belgium, France, Germany and Sweden. Prev Vet Med. (2016) 130:41–50. 10.1016/j.prevetmed.2016.06.003 27435645

[34] Hopkins D Poljak Z Farzan A Friendship R. Factors contributing to mortality during a *Streptoccocus suis* outbreak in nursery pigs. Can Vet J. (2018) 59:623–30. 29910476 PMC5949957

[35] Correa-Fiz F Neila-Ibáñez C López-Soria S Napp S Martinez B Sobrevia L. Feed additives for the control of post-weaning Streptococcus suis disease and the effect on the faecal and nasal microbiota. Sci Rep. (2020) 10:20354. 10.1038/s41598-020-77313-6 33230191 PMC7683732

[36] Cho JG Dee SA. Porcine reproductive and respiratory syndrome virus. Theriogenology. (2006) 66:655–62. 10.1016/j.theriogenology.2006.04.024 16730057

[37] Saade G Deblanc C Bougon J Marois-Créhan C Fablet C Auray G. Coinfections and their molecular consequences in the porcine respiratory tract. Vet Res. (2020) 51:80. 10.1186/s13567-020-00807-8 32546263 PMC7296899

[38] Burch DGS Sperling D. Amoxicillin-current use in swine medicine. J vet Pharmacol Therap. (2018) 41:356–68. 10.1111/jvp.12482 29352469

[39] Voorspoels J D'Haese E de craene BA Vervaet C de Riemaecker D Deprez P. Pharmacokinetics of florfenicol after treatment of pigs with single oral or intramuscular doses or with medicated feed for three days. Vet Rec. (1999) 145:397–9. 10.1136/vr.145.14.397 10574273

[40] Li X Xie S Pan Y Qu W Tao Y Chen D. Preparation, characterization and pharmacokinetics of doxycycline hydrochloride and florfenicol polyvinylpyrroliddone microparticle entrapped with hydroxypropyl-□-cyclodextrin inclusion complexes suspension. Colloids Surf B Biointerfaces. (2016) 141:634–42. 10.1016/j.colsurfb.2016.02.027 26918512

[41] Ling Z Yonghong L Junfeng L Li Z Xianqiang L. Tilmicosin- and florfenicol-loaded hydrogenated castor oil-solid lipid nanoparticles to pigs: Combined antibacterial activities and pharmacokinetics. J vet Pharmacol Therap. (2018) 41:307–13. 10.1111/jvp.12465 29139136

[42] Holmer I Salomonsen CM Jorsal SE Astrup LB Jensen VF Høg BB. Antibiotic resistance in porcine pathogenic bacteria and relation to antibiotic usage. BMC Vet Res. (2019) 15:449. 10.1186/s12917-019-2162-8 31829171 PMC6907208

[43] Winkelman NL Crane JP Elfring GD Dal Kratzer D Meeuwse DM Dame KJ. Lincomycin-medicated feed for the control of porcine proliferative enteropathy (ileitis) in swine. J Swine Health Product. (2002) 10:107–11.

[44] Alexopoulos C Tassis PD Kyriakis CS Tzika ED Papatsiros V Kyriakis SC. First experience on the effect of in-feed lincomycin for the control of proliferative enteropathy in growing pigs. J Vet Med Series A. (2006) 53:157–62. 10.1111/j.1439-0442.2006.00803.x 16533333

[45] Zhang B Ku X Yu X Sun Q Wu H Chen F. Prevalence and antimicrobial susceptibilities of bacterial pathogens in Chinese pig farms from 2013 to 2017. Sci Rep. (2019) 9:9908. 10.1038/s41598-019-45482-8 31289289 PMC6616368

[46] WHO. Critically Important Antimicrobials for Human Medicine. 6th Revision 2018. Ranking of Medically Important Antimicrobials for Risk Management of Antimicrobial Resistance Due to Non-human Use. WHO (2018).

[47] Bolinger H Kathariou S. The current state of macrolide resistance in campylobacter spp.: trends and impacts of resistance mechanisms. Appl Environ Microbiol. (2017) 83:e00416-17. 10.1128/AEM.00416-17 28411226 PMC5452823

[48] Scali F Santucci G Maisano AM Giudici F Guadagno F Tonni M. The use of antimicrobials in italian heavy pig fattening farms. Antibiotics. (2020) 9:892. 10.3390/antibiotics9120892 33322049 PMC7764202

[49] Du T Nan Y Xiao S Zhao Q Zhou E-M. Antiviral strategies against PRRSV infection. Trends Microbiol. (2017) 25:968–79. 10.1016/j.tim.2017.06.001 28652073

[50] Bolhuis JE Schouten WGP Schrama JW Wiegant VM. Behavioural development of pigs with different coping characteristics in barren and substrate-enriched housing conditions. Appl Anim Behav Sci. (2005) 93:213–28. 10.1016/j.applanim.2005.01.006

[51] van Dixhoorn IDE Reimert I Middelkoop J Bolhuis JE Wisselink HJ Groot Koerkamp PWG. Enriched housing reduces disease susceptibility to co-infection with Porcine Reproductive and Respiratory Virus (PRRSV) and Actinobacillus pleuropneumoniae (A. pleuropneumoniae) in young pigs. PLoS ONE. (2016) 11:e0161832. 10.1371/journal.pone.0161832 27606818 PMC5015855

[52] Merle R Robanus M Hegger-Gravenhorst C Mollenhauer Y Hajek P Käsbohrer A. Feasibility study of veterinary antibiotic consumption in Germany - comparison of ADDs and UDDs by animal production type, antimicrobial class and indication. BMC Vet Res. (2014) 10:7. 10.1186/1746-6148-10-7 24401194 PMC3895796

[53] Kasabova S Hartmann M Werner N Käsbohrer A Kreienbrock L. Used daily dose vs. defined daily dose—contrasting two different methods to measure antibiotic consumption at the farm level. Front Vet Sci. (2019) 6:116. 10.3389/fvets.2019.00116 31069237 PMC6491814

